# A Mouse Model of L-2-Hydroxyglutaric Aciduria, a Disorder of Metabolite Repair

**DOI:** 10.1371/journal.pone.0119540

**Published:** 2015-03-12

**Authors:** Rim Rzem, Younes Achouri, Etienne Marbaix, Olivier Schakman, Elsa Wiame, Sandrine Marie, Philippe Gailly, Marie-Françoise Vincent, Maria Veiga-da-Cunha, Emile Van Schaftingen

**Affiliations:** 1 Welbio and Laboratory of Physiological Chemistry, de Duve Institute, Université catholique de Louvain, Brussels, Belgium; 2 Cell Unit, de Duve Institute, Université catholique de Louvain, Brussels, Belgium; 3 Laboratory of Cell Physiology, Institute of Neuroscience, Université catholique de Louvain, Brussels, Belgium; 4 Laboratory of Metabolic Diseases, Cliniques Universitaires Saint-Luc, Université catholique de Louvain, Brussels, Belgium; The University of Iowa, UNITED STATES

## Abstract

The purpose of the present work was to progress in our understanding of the pathophysiology of L-2-hydroxyglutaric aciduria, due to a defect in L-2-hydroxyglutarate dehydrogenase, by creating and studying a mouse model of this disease. L-2-hydroxyglutarate dehydrogenase-deficient mice (*l2hgdh* ^-/-^) accumulated L-2-hydroxyglutarate in tissues, most particularly in brain and testis, where the concentration reached ≈ 3.5 μmol/g. Male mice showed a 30% higher excretion of L-2-hydroxyglutarate compared to female mice, supporting that this dicarboxylic acid is partially made in males by lactate dehydrogenase C, a poorly specific form of this enzyme exclusively expressed in testes. Involvement of mitochondrial malate dehydrogenase in the formation of L-2-hydroxyglutarate was supported by the commensurate decrease in the formation of this dicarboxylic acid when down-regulating this enzyme in mouse *l2hgdh* ^-/-^ embryonic fibroblasts. The concentration of lysine and arginine was markedly increased in the brain of *l2hgdh* ^-/-^ adult mice. Saccharopine was depleted and glutamine was decreased by ≈ 40%. Lysine-α-ketoglutarate reductase, which converts lysine to saccharopine, was inhibited by L-2-hydroxyglutarate with a Ki of ≈ 0.8 mM. As low but significant activities of the bifunctional enzyme lysine-α-ketoglutarate reductase/saccharopine dehydrogenase were found in brain, these findings suggest that the classical lysine degradation pathway also operates in brain and is inhibited by the high concentrations of L-2-hydroxyglutarate found in *l2hgdh* ^-/-^ mice. Pathological analysis of the brain showed significant spongiosis. The vacuolar lesions mostly affected oligodendrocytes and myelin sheats, as in other dicarboxylic acidurias, suggesting that the pathophysiology of this model of leukodystrophy may involve irreversible pumping of a dicarboxylate in oligodendrocytes. Neurobehavioral testing indicated that the mice mostly suffered from a deficit in learning capacity. In conclusion, the findings support the concept that L-2-hydroxyglutaric aciduria is a disorder of metabolite repair. The accumulation of L-2-hydroxyglutarate exerts toxic effects through various means including enzyme inhibition and glial cell swelling.

## INTRODUCTION

L-2-hydroxyglutaric aciduria is an autosomal recessive neurometabolic disorder characterized by the accumulation of L-2-hydroxyglutarate [1, 2, 3, 4] and caused by a deficiency in L-2-hydroxyglutarate dehydrogenase [5, 6, 7]. This non-classical metabolite, which does not belong to any conventional metabolic pathway in eukaryotes, appears to be formed in most tissues as the result of a side activity of L-malate dehydrogenase, principally its mitochondrial form. This enzyme, which is abundant in tissues, catalyses the reduction of oxaloacetate to L-2-hydroxyglutarate with a catalytic efficiency that is about 10^7^ times lower than that with which it catalyses the reduction of oxoloacetate to L-malate [8]. Another potential source of L-2-hydroxyglutarate is lactate dehydrogenase C (also called lactate dehydrogenase X), a form of lactate dehydrogenase that is exclusively expressed in testes, and which acts not only on pyruvate, as do the lactate dehydrogenases encoded by the LDHA and LDHB gene, but also on other α-ketoacids including α-ketoglutarate, thus producing, at least *in vitro*, L-2-hydroxyglutarate (reviewed by [9]). There is no known role for L-2-hydroxyglutarate and its concentration is normally maintained at a very low value in tissues thanks to L-2-hydroxyglutarate dehydrogenase, an FAD-linked, mitochondrial enzyme [5, 7, 10]. This observation led to the idea that L-2-hydroxyglutarate dehydrogenase serves a metabolite repair role, by destroying a useless metabolite, formed because of the lack of absolute specificity of mitochondrial L-malate dehydrogenase. This and other examples of enzymes playing a metabolite repair role have recently been reviewed [11, 12].

Little is presently known on the cause of the toxicity of L-2-hydroxyglutarate. Its structural resemblance to L-glutamate and α-ketoglutarate, two major metabolites, makes it likely that the pathophysiology of the disease involves inhibition of enzymes acting normally on glutamate or α-ketoglutarate, or even abnormal utilization of L-2-hydroxyglutarate by such enzymes. Testing such hypothesis requires knowledge on the biochemical perturbations induced by the accumulation of L-2-hydroxyglutarate.

Here, we created a mouse model of L-2-hydroxyglutaric aciduria to progress in our understanding of the pathophysiology of this disease, and most particularly to identify the potential biochemical and pathophysiological consequences of L-2-hydroxyglutarate accumulation.

## MATERIALS and METHODS

### Animals and ethics statement

All mice were raised under standard husbandry conditions and were housed in colony cages with a 12h light/dark cycle. They had free access to water and food, unless otherwise specified. The experiments were performed with the approval of the Ethics Committee on Animal Experiments of the Catholic University of Louvain—department of health sciences (permit number: 2013/UCL/MD/015). Animals were euthanized with CO_2_ immediately before removing the tissues (biochemical analysis) or deeply anesthetized with pentobarbital (histological and ultrastructural analysis) to minimize suffering.

### Generation of the knockout mice

**Fig 1:**
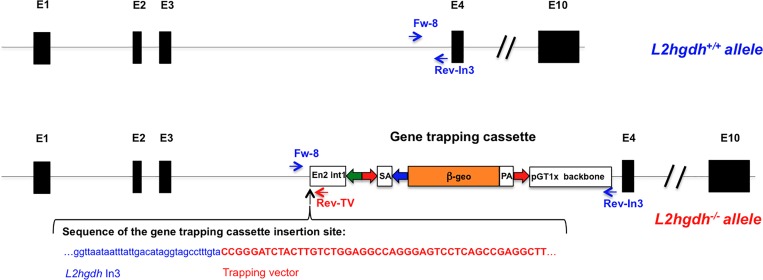
Generation of the murine *l2hgdh* ^-/-^ model by insertion of a gene trapping cassette. We show the genomic structure of the *l2hgdh* gene in wild-type and knock-out mice, which spans 10 exons (black boxes). In the disrupted allele, the gene trapping cassette [containing engrailed intron1 (En2 int1); Lox71 and LoxP Cre recombination sites (green and blue arrows); FRT flippase recombination sites (red arrows); β-geo cassette (orange box); splicing acceptor (SA) and polyadenylation (PA) sequences; a portion of pGTX1 vector (pUC backbone)] has been randomly inserted in intron 3, which leads to the production of an inactive protein comprising the first 137 residues of L-2-hydroxyglutarate dehydrogenase fused to the β-geo cassette. The latter confers resistance to neomycin and interrupts downstream transcription due to the addition of a polyadenylation signal. The precise site of insertion of the gene-trapping cassette is shown, as well as the location of the primers (Fw-8; Rev-In3; Rev-TV) used for diagnostic amplification of the wild-type and mutated alleles.

Mouse embryonic stem cells (cell lineE14Tg2A.4, strain 129/Ola) containing an insertional mutation in the *l2hgdh* gene [13] were obtained from MMRRC (Mutant Mouse Regional Resource Centers funded by NIH). The gene-trap vector used (pGT1x) contains a splice-acceptor sequence upstream of a reporter gene, β-geo (a fusion of β-galactosidase and neomycin phosphotransferase II). The insertional mutation occurred in the third intron. Thus the mutation resulted in the production of a fusion transcript encoding the first 137 aminoacid residues of L-2-hydroxyglutarate dehydrogenase followed by β-geo. Ten to 15 embryonic stem cells were injected into the blastocoele of C57BL/6 blastocysts. Forty injected embryos were incubated in KSOM medium [14] at 37°C for at least 3 hours before being transferred into oviducts of CD1 pseudopregnant females. Ten chimeric males were obtained. To test the capacity of embryonic stem cells to contribute to the germline, the male chimeras were mated with C57BL/6 females. Germline transmission of the embryonic stem cells genotype was indicated by the production of agouti offspring and confirmed by genotyping by PCR-analysis of tail DNA. The presence of the wild-type (*l2hgdh* ^*+/+*^) and mutated alleles (*l2hgdh* ^-/-^) was detected in specific PCR-amplifications using a common forward primer (Fw-8: 5’-TTTCTGAGTTCGAGGTCAGCCTG-3’; see Fig. 1) and two downstream reverse ones. These were either in intron 3 of the *l2hgdh* gene (Rev-In3: 5’-CAGAATTCCATGAAGTGGTAC-3’) or in the inserted DNA (Rev-TV: 5’-GGACTCCCTGGCCTCCAG −3’). The length of the two amplification products was approximately 300 bp. All mice described herein had a mixed genetic background (~ 50% C57BL/6 and ~ 50% 129 Ola). All studied *l2hgdh* ^*+/+*^ and *l2hgdh* ^-/-^ mice were generated by crossing heterozygous mice.

### Amino acid analysis

Mice starved for 24 h were euthanized as described above, blood was collected, brain and testes were rapidly removed and frozen in liquid nitrogen. Frozen samples were homogenized with two vol. of 15% sulfosalicylic acid. Heparinized plasma was mixed with one vol. of 10% sulfosalicylic acid. After centrifugation (16000 x *g*; 15 min at 4°C), the amino acids of plasma and of brain and testes homogenates were separated and quantified using a Biochrom 30 amino acids analyzer. For the assay of cystine, frozen brain (0.2 g) was homogenized with two volumes (0.4 ml) of 5.2 mM N-ethylmaleimide. Following homogenization, the homogenate was supplemented with 0.12 ml of 15% sulfosalicylic acid and clarified by centrifugation (16000 x *g*; 15 min at 4°C). Cystine content was measured by LC/MS/MS using an isotopic dilution method [15]. Pipecolate was determined as described in [16].

### L-2-hydroxyglutarate assay

For the measurement of relatively high concentrations of L-2-hydroxyglutarate (as in tissues and urine of *l2hgdh* ^-/-^ mice), we used a radiochemical assay that was based on the competition exerted by unlabeled L-2-hydroxyglutarate on the use of radiolabelled L-2-hydroxyglutarate by partially purified L-2-hydroxyglutarate dehydrogenase [5, 17]. Tissues were directly homogenized in 2 vol. of 10% (v/v) HClO_4_. After centrifugation (16 000 x *g* for 10 min at 4°C), the supernatant was neutralized with 3M KHCO_3_. Where indicated, (D+L)-2-hydroxyglutarate was assayed in heated tissue extracts by GC/MS. The samples were treated with hydroxylamine hydrochloride in the presence of pentadecanoic acid as an internal standard, and extracted with ethyl acetate followed by diethyl ether. After evaporation of the combined extracts under a stream of nitrogen, derivatization was achieved with N,O-bis(trimethylsilyl)trifluoroacetamide:trimethylchlorosilane (BSTFA:TMCS; 99:1). Analysis was performed by GC/MS using a Hewlett-Packard 6890 series GC system equipped with 30 m x 0.25-mm fused silica capillary column CP-SIL 8 CB from Varian, with helium as carrier gas. Detection was performed with a Hewlett-Packard 5973 mass-selective detector under electron impact fragmentation and with the scan and selective ion monitoring modes for acquisition. To identify and quantitate 2-hydroxyglutarate, we compared its retention time, mass spectra and detector response to those of the commercial compound.

### siRNA transfection of MEF primary cell cultures

Mouse embryonic fibroblasts (MEF) isolated from *l2hgdh* ^*+/+*^ and *l2hgdh* ^-/-^ mice embryos were maintained and transfected with siRNAs as described [18]. Control siRNA or mitochondrial malate dehydrogenase siRNA [both from Dharmacon ON-TARGETplus SMARTpool from Thermo Scientific (Epsom, UK)] was added at a final concentration of 50 nM using Lipofectamine 2000 (Invitrogen) as the transfection reagent. After 48 h, protein was extracted from cells. The cells were washed with 1 ml phosphate buffered saline (PBS) and harvested in 100 μl of 20 mM Hepes (pH 7.1) containing 5 μg/ml antipain as described [7]. Cell media (1 ml) were used to determine the concentration of 2-hydroxyglutarate by gas chromatography analysis. The activity of mitochondrial malate dehydrogenase was determined in cell extracts [8].

### Western blot analysis

Equal amounts (20 μg) of cell extracts were analyzed by SDS-PAGE. Proteins were transferred to a Hybond-C Extra membrane (GE Healthcare, Roosendaal, The Netherlands). Next, the membrane was incubated for 1 h at room temperature with 5% skimmed milk in Tris-buffered saline followed by an overnight incubation at 4°C with monoclonal anti-mitochondrial malate dehydrogenase antibody (Human Protein Atlas HPA019714, 1:500) in the same buffer. The western blot was further performed according to the manufacturer’s protocol.

### Partial purification and assay of lysine -α-ketoglutarate reductase

Mouse liver or brain was homogenized with 4 volumes of a buffer containing 25 mM Hepes, pH 7.1, 25 mM KCl, 2 μg/ml leupeptin and antipain. The homogenate was centrifuged at 15 000 x *g* for 15 min at 4°C and 1 ml of the supernatant was applied on a Blue Trisacryl column (1 ml bed volume) equilibrated in 20 mM Hepes, pH 7.1. The column was washed with 1 ml of the equilibration buffer and protein was eluted with a stepwise NaCl gradient (0.1, 0.2, 0.5, 1.0, 1.5 and 2.0 M; 1 ml/step) in the same buffer. The enzyme activity was assayed spectrophotometrically at 30°C in the presence of 25 mM Hepes, pH 8.0, 0.15 mM NADPH, 1 mM dithiothreitol, 5 mM α-ketoglutarate and 10 mM lysine. Saccharopine dehydrogenase was assayed in the presence of the same buffer, 0.5 mM NAD^+^, and 0.5 mM saccharopine.

### RNA isolation and Q-PCR analysis

Total RNA was extracted from tissues as previously described [18]. RNA concentrations were determined by measuring the absorbance at 260 nm. To determine specific mRNA levels, cDNA synthesis and quantitative real-time PCR were performed as described previously [18] using RevertAid H minus reverse transcriptase (Thermo Scientific) and SYBER Green fluorescent dye (Bio-Rad), respectively [19]. The sequence of primers used for amplification can be obtained upon request.

### Histological and ultrastructural analysis

Brain histological analysis was performed in mice of various ages, but most of the data presented here derives from analyses performed on *l2hgdh* ^-/-^ and *l2hgdh* ^*+/+*^ 5 to 7 month old littermates (3 males and 4 females from each genotype). The mice were deeply anesthetized with pentobarbital and trans-cardially perfused with phosphate buffered saline followed by whole-body perfusion-fixation with 10% neutral-buffered formalin through the left ventricle under a height of 120 cm, after right atrium excision for outlet, as described [20]. The brain was quickly dissected. A transverse median slice of the brain of male mice was immediately immersed in 0.5% glutaraldehyde for ultrastructural analysis. The rest of the brain and the whole brain of female mice were immersed in formalin overnight. A transverse median slice of female brain was then put in O. C. T. medium (Sakura, Alphen aan den Rijn, The Netherlands) and frozen in liquid nitrogen-cooled isopentane. The other slices were embedded in paraffin. The spinal cord of the female mice was similarly dissected, fixed and embedded in paraffin.

For light microscopy, histological sections were stained with hematoxylin and eosin, alcian blue, or luxol fast blue. Frozen sections were stained with oil red O or Sudan black. Immunohistochemical analysis of a neuron-specific nuclear protein (with monoclonal mouse anti-NeuN IgG_1_ antibody, clone A60; Millipore, Bedford, MA; used at 5 μg/ml) and of oligodendrocyte transcription factor 2 (with antigen affinity-purified polyclonal goat IgG anti-human Olig 2; R&D Systems, Abingdon, UK; used at 0.6 μg/ml) was performed using the ultraView Universal diaminobenzidine detection kit on a Ventana Benchmark XT module (Roche, Basel, Switzerland). Immunohistochemical analysis of Ki67 (monoclonal mouse IgG_1_K, clone B56; BD Pharmingen, Erembodegem, Belgium; used at 5 μg/ml) was performed after antigen retrieval by boiling deparaffinized sections for 75 min. Cleaved caspase-3 (polyclonal rabbit antibodies, from Cell Signaling—Millipore; 1:100) was immunodetected without antigen retrieval. After incubation overnight at 4°C with the primary antibody, sections were rinsed, incubated with peroxidase-conjugated dextran molecules carrying anti-mouse or rabbit IgG antibodies (Envision, DakoCytomation, Glostrup, Denmark), followed by incubation with H_2_O_2_ and diaminobenzidine. Mouse small bowel was used as positive control for the latter two antigens.

For ultrastructural analysis, tissue pieces were post-fixed in 1% OsO_4_ in 0.1 M cacodylate buffer for 1 h, rinsed in veronal buffer and stained overnight “en bloc” in 1% neutral uranyl acetate in the dark, all at 4°C. After extensive washing in veronal buffer, blocks were dehydrated in graded ethanol and embedded in Spurr. Areas for ultrathin sections were selected on toluidine blue stained semi-thin sections. Ultrathin (70 nm nominal) sections were obtained with a Reichert ultramicrotome (Reichert, Austria), collected on rhodanium 400 mesh grids and contrasted with 3% uranyl acetate followed by lead citrate for 10 min each. Grids were washed with water, dried and examined in a FEI CM12 electron microscope (Philips, Eindhoven, The Netherlands) operating at 80 kV.

### Neurobehavioral testing

#### Modified SHIRPA test

This test was used as a simple first-line observational phenotypic test [21]. Briefly, the mice are run through a battery of tests consisting in the observation of a variety of spontaneous (viewing jar) and provoked behaviors (arena) such as body position, rearing, locomotor activity, gait, reflexes, and anxiety. A score sheet was used to record the observation. A description and list of the scoring parameters can be found at http://empress.har.mrc.ac.uk/.

#### Open-Field Test

This test was used to assess nonforced ambulation in a new environment as mice can move freely without any influence of the examiner. Briefly, mice were placed twice at one-month interval in a square arena (60 x 60 cm) and video-tracked (Ethovision 6.1, Noldus; Wageningen, The Netherlands) for 20 min. The total distance covered by the animals was measured [22].

#### Grip Strength Test

Combined forelimb and hindlimb muscle strength was measured by gently lowering a mouse on the top of a grid connected to a sensor (Panlab-Bioseb, Vitrolles France) so that both front paws and hind paws could grip the grid. The mice were then pulled back steadily down the complete length of the grid until the grip was released. This test consisted of three trials performed with 15 min intervals. Results are means of the two highest values of force recorded, related to body weight [22].

#### Rotarod test

Mice were tested for their ability to keep their balance and coordination on a rotating rod (Bioseb, Vitrolles, France). The time (latency) taken to fall off the rod rotating under continuous acceleration (e.g. from 4 to 40 rpm) was measured. Mice performed 3 trials per day during 5 days. Animals staying for 300 s were taken from the rotarod and recorded as 300 s. Results are presented as the mean of the 3 trials [23].

#### Y maze test

This test was used to assess working memory. The Y maze was made of 3 identical opaque arms (35 cm length x 5 cm width x 10 cm height) placed at 120° from each other. Mice were placed into a start arm and video-tracked with Ethovision 6.1 for 5 min. Total number of arm entries and arm alternation were recorded. Alternations (taken as an indicator of short-term memory) are determined as successive entries into three different arms.

#### Water maze test

The Morris water maze test was used to assess spatial learning and memory. Water maze was made of a round pool with a diameter of 113 cm, virtually divided into four quadrants (North, South, West and East) and filled with water (26°C). Several visual cues were placed around the pool. Mice were tested on 5 consecutive days with 5 consecutive trials per day. During the trials of the first 4 days, the animals were placed in the pool facing the sidewall and allowed to swim freely to the platform. The latter was placed at the center of the North-West quadrant of the pool and was visible on the training day and submerged 1 cm under the water surface on the 3 following days. The initial position in which the animal was left in the pool varied among trials. If the animal did not find the platform during a 60 s period, it was gently guided to it, allowed to remain on the platform for 5 s, and removed from the pool before being placed in the next initial starting position in the pool. On the 5^th^ day, the animals were submitted to a probe test, which consisted in allowing the mice to swim freely for 60 s in the pool from which the escape platform had been removed. During all these tests, the mice were video-tracked with Ethovision 6.1. The escape latency time (i.e. the time to reach the platform), the swimming speed, the time spent in each quadrant and the distance from the platform location were registered [24].

#### Metabolic analyses

Mice basal activity (rearing and locomotor activity), food and water intake were measured in individual ‘Physiocages’ (Panlab-Bioseb, Vitrolles, France) during 48 h after 24 h of habituation [25].

#### Wire test

Mice were suspended by their forelimbs to a 1.5-mm-thick, 60-cm-long metallic wire 45 cm above soft ground. The time of latency until the mouse completely released its grasp and fell down was recorded. Three trials were performed per session, with a 30-s recovery period between trials. The maximum time per trial was set to 180 s. For each mouse, the latency times of the three trials were averaged [26].

## RESULTS

### Generation of *l2hgdh* ^-/-^ mice

ES cells with a genetrap inactivation of the *l2hgdh* gene were obtained from the gene trap consortium. Sequencing of the chimeric cDNA indicated that the vector had been inserted in intron 3 (Fig. 1) leading to a fusion protein comprising the first 137 residues of L-2-hydroxyglutarate dehydrogenase fused to a β-geo cassette. Downstream transcription of the *l2hgdh* gene is interrupted by a polyadenylation signal present in the genetrap vector. The precise site of insertion was determined by PCR amplification and sequencing (see Fig. 1). This allowed the design of primers for the specific amplification of DNA for the mutated and the non-mutated alleles of the gene (see Materials and Methods).

Male chimeras obtained as described in the Materials and Methods were mated with C57BL/6 mice to found a colony. The crossing of heterozygous mice yielded proportions of *l2hgdh* ^*+/+*^, *l2hgdh* ^*+/-*^, and *l2hgdh* ^-/-^ (66:125:64) close to the expected 25:50:25 proportion, indicating no embryonic lethality of the *l2hgdh* ^-/-^ mice.

qPCR experiments on liver mRNA with primers in exon 4 and exon 5 of the *l2hgdh* transcript indicated that the mRNA was nearly absent (<5% of the normal value) in *l2hgdh* ^-/-^ mice and amounted to ≈ 50% of the normal value in heterozygous mice (not shown). The finding of low but detectable levels of transcripts in *l2hgdh* ^-/-^ mice indicates that the polyadenylation signal present in the inactivation cassette is leaky. The presence of potential normal transcripts, due to splicing of exon 3 with the normal exon 4 was tested by quantitative RT-PCR amplification of the cDNA with primers hybridizing with these two exons. Starting from brain cDNA from *l2hgdh* ^-/-^ mice, we observed an amplification product of the same size as with cDNA obtained from *l2hgdh* ^*+/+*^ mice. qPCR indicated, however, that its quantity in *l2hgdh* ^-/-^ mice amounted to ≈ 0.67 ± 0.15% (mean ± SEM for n = 8) of the amount found in *l2hgdh* ^*+/+*^ mice. These findings suggest that in *l2hgdh* ^-/-^ mice, a small amount of readthrough transcript is synthesized and spliced in a similar manner as the wild type RNA. However, the resulting L-2-hydroxyglutarate dehydrogenase activity should represent no more than 1% of the normal activity.

There was no obvious difference in lethality between *l2hgdh* ^*+/+*^ and *l2hgdh* ^-/-^ mice for the animals kept in the specific-pathogen-free animal house, but there was a tendency to a higher lethality (5/23 as compared to 0/24) when the mice were taken out of this environment (e.g. for testing).

Since male *ldhx* ^-/-^ mice are hypofertile [27], we tested the fertility of male *l2hgdh* ^-/-^ mice, but did not find evidence for decreased fertility. Indeed, crossing knockout male and female mice gave litters of similar size to the ones resulting from the crossing of wild-type mice.

### L-2-hydroxyglutarate: levels in tissues and origin

**Fig 2:**
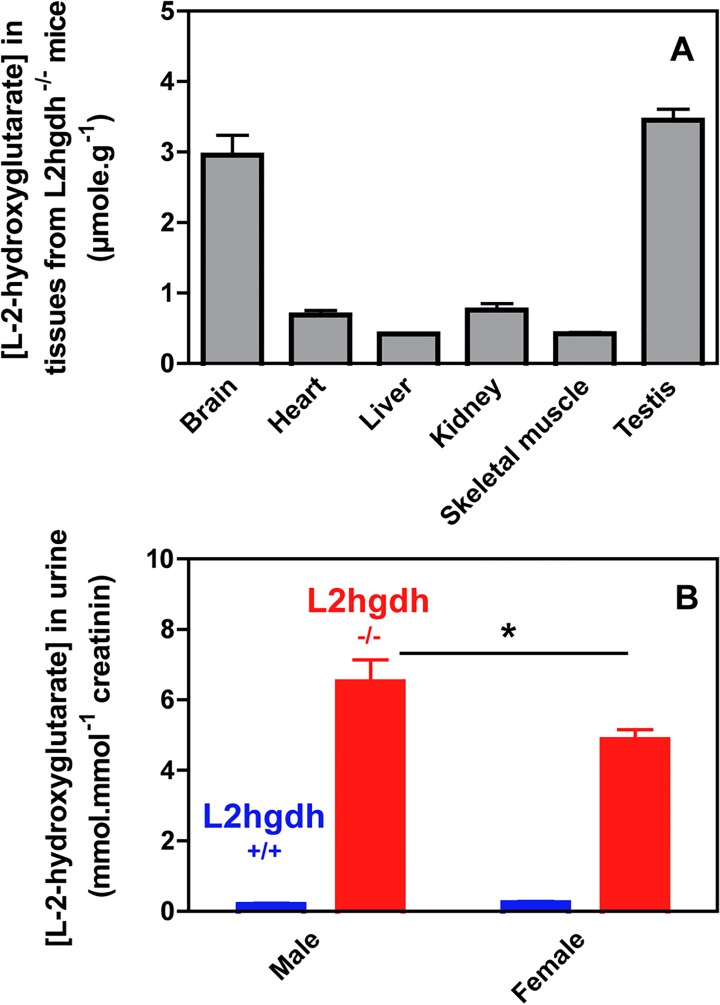
L-2-hydroxyglutarate concentration in different tissues (A) and its urinary excretion (B) in *l2hgdh* ^*+/+*^ and *l2hgdh* ^-/-^ mice. Tissues of *l2hgdh* ^-/-^ male mice (n = 4) were analyzed in (A). Values of urinary excretion for male and female mice (n = 3) are shown in (B). Results are means ± SEM. * p < 0.05 by unpaired t test.

Analysis of L-2-hydroxyglutarate in tissues of *l2hgdh* ^-/-^ mice (Fig. 2A) using our bioassay indicated that this compound was particularly abundant in brain and in testis, where its concentration amounted to ≈ 3.5 μmol/g, with lower values in other tissues. L-2-hydroxyglutarate was undetectable in tissues from normal mice (< 100 μM with the bioassay), to the exception of testis, where its concentration amounted to ≈ 0.35 μmol/g in control mice (not shown). L-2-hydroxyglutarate levels were also determined in 24 h urine collections. As shown in Fig. 2B, urinary excretion of L-2-hydroxyglutarate amounted to 6.5 ± 0.6 and 4.9 ± 0.3 mol/mol creatinine in male and female *l2hgdh* ^-/-^ mice, respectively, indicating that male mice produced about 30% more L-2-hydroxyglutarate than female mice, presumably due to the activity of the testis-specific enzyme lactate dehydrogenase C on α-ketoglutarate (see Discussion).

**Fig 3:**
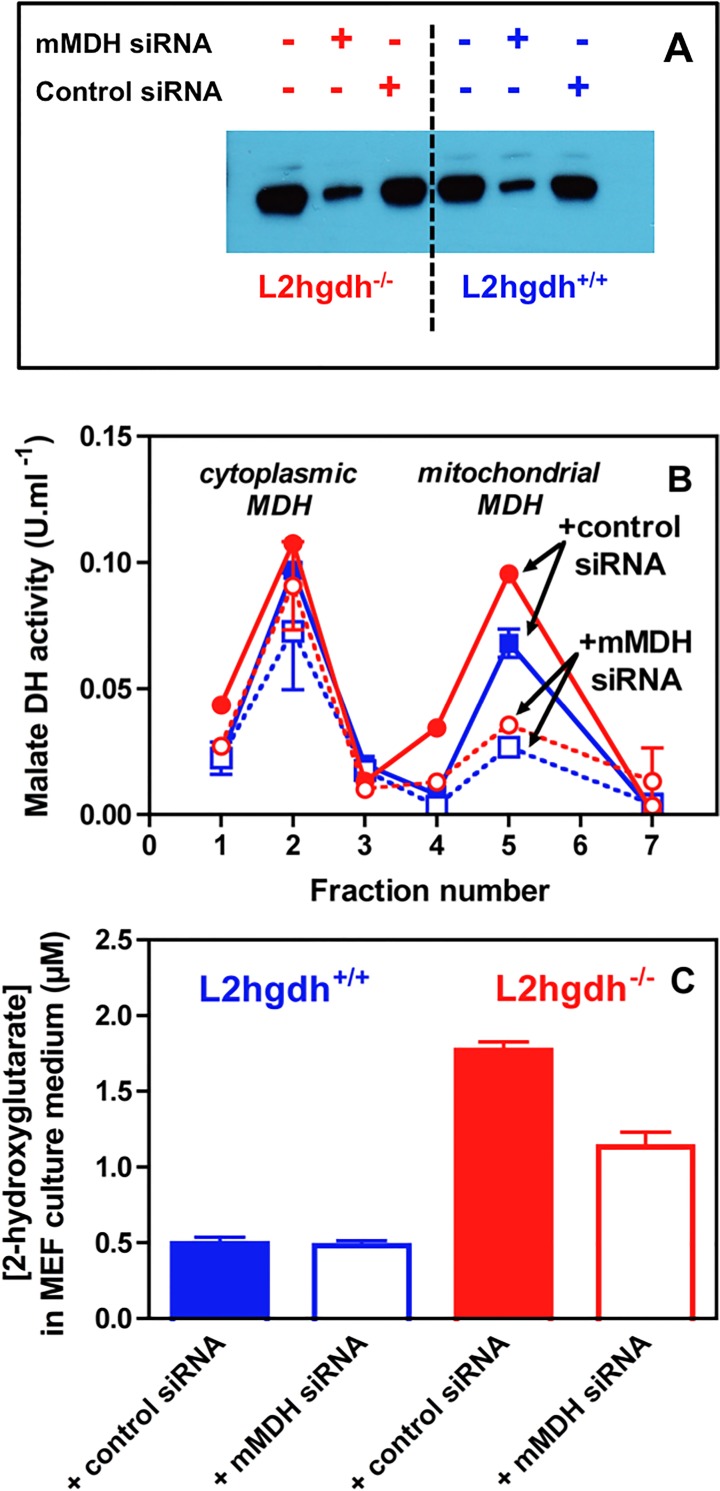
L-2-hydroxyglutarate formation in *l2hgdh* ^-/-^ cells is decreased if mitochondrial malate dehydrogenase (mMDH) is knocked down. MEF cells derived from *l2hgdh* ^*+/+*^ and *l2hgdh* ^-/-^ 14.5 day-embryos were treated with control (scrambled) or mMDH-specific siRNAs for 48 h. (A) mMDH immunoreactivity was determined in lysates of cells treated or not with siRNA. (B) mMDH activity in cell lysates was determined by separating this enzyme from cytoplasmic MDH by cation exchange chromatography. (C) (D+L)-2-hydroxyglutarate concentration was determined in the medium by GC-MS.

**Fig 4:**
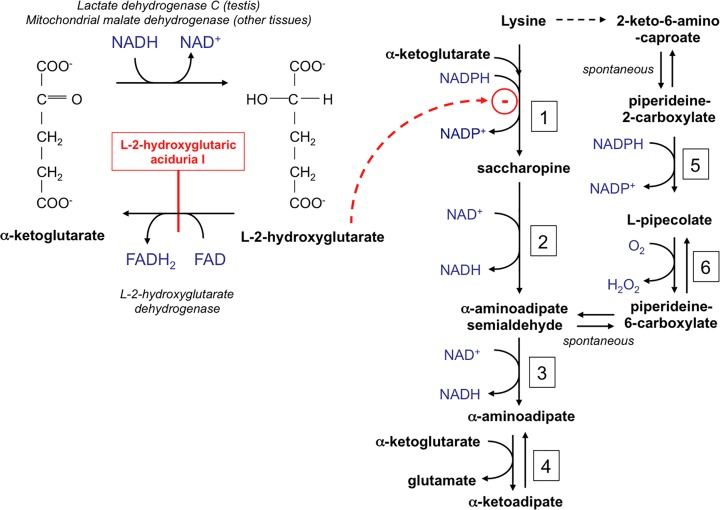
Formation and breakdown of L-2-hydroxyglutarate and interference of this compound with lysine metabolism. The scheme shows how L-2-hydroxyglutarate is formed and degraded. It also shows the initial steps of the major lysine catabolic pathway (via saccharopine) present in mammalian tissues and of the minor pathway (via L-pipecolate) present in brain and the inhibition exerted by L-2-hydroxyglutarate. 1. Lysine-α-ketoglutarate reductase; 2. Saccharopine dehydrogenase; 3. α-Aminoadipate semialdehyde dehydrogenase; 4. α-Aminoadipate transaminase; 5. Imine reductase; 6. L-Pipecolate oxidase.

Previous work has shown that the L-2-hydroxyglutarate-producing enzyme co-elutes with mitochondrial L-malate dehydrogenase in liver [8]. To verify that this enzyme is the main one involved in L-2-hydroxyglutarate production in tissues other than testis, we investigated the effect of mitochondrial L-malate dehydrogenase suppression on the accumulation of L-2-hydroxyglutarate in *l2hgdh* ^-/-^ MEF (mouse embryonic fibroblasts) cells (Fig. 3). Treatment of these cells with siRNAs specific for mitochondrial L-malate dehydrogenase caused an almost complete disappearance of the mitochondrial L-malate dehydrogenase mRNA and a partial disappearance of immunoreactive mitochondrial L-malate dehydrogenase (Fig. 3A) and enzymatic activity (Fig. 3B). Consistent with a partial decrease in mitochondrial L-malate dehydrogenase activity, we found that (D+L)-2-hydroxyglutarate concentration in the cell culture media was reduced by about 2-fold in *l2hgdh* ^-/-^ MEF cells (Fig. 3C). The (D+L)-2-hydroxyglutarate concentration was not affected by knockdown of mitochondrial L-malate dehydrogenase in *l2hgdh* ^*+/+*^ cells, indicating that mitochondrial L-malate dehydrogenase specifically affected the production of the L-isomer of 2-hydroxyglutarate. Taken together these findings indicate that the main two enzymes involved in L-2-hydroxyglutarate formation are mitochondrial L-malate dehydrogenase and lactate dehydrogenase C (see Fig. 4).

### Modification of amino acids in brain

**Fig 5:**
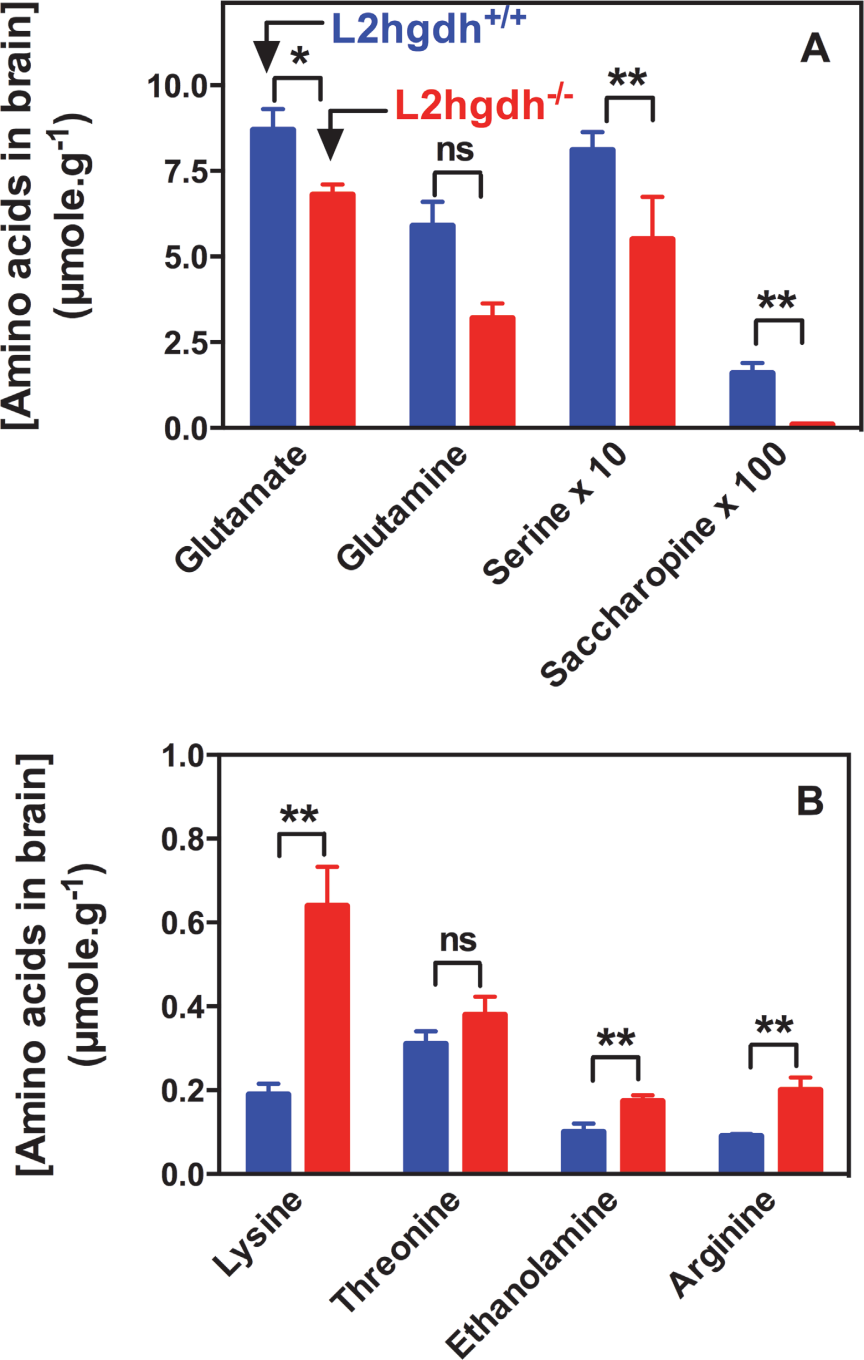
Decreased (A) or increased (B) concentrations of brain amino acids of *l2hgdh* ^-/-^ mice compared to *l2hgdh* ^*+/+*^ mice. Brains from 24 h fasted mice aged 4–6 months were analyzed. ‘Serine x 10’ and ‘Saccharopine x 100’ indicate that the actual concentrations in brain is 10-fold or 100-fold lower, respectively, compared to the values that are shown in the Y axis. Results are the means ± SEM for n = 10 (*l2hgdh* ^*+/+*^) or n = 6 (*l2hgdh* ^-/-^) mice. *: 0.0003 < alpha < 0.0016; **: alpha < 0.0003; ns: non significant. The analysis was performed using the Student t-test with a Bonferroni correction for multiple testing (see also Table 1).

Analysis of amino acids and other amines in brain showed a marked increase in the concentration of lysine (≈ 3.4-fold), arginine (2.3-fold), and free ethanolamine (1.7 fold). Saccharopine was depleted, and glutamine and glutamate were decreased by 45 and 22%, respectively (Fig. 5 and Table 1). Saccharopine, cystine and pipecolate are difficult to separate with the conventional technique used for the determination of amino acids. However, close inspection of the chromatograms of samples enriched with standards indicated that the peak that we attributed to saccharopine was precisely eluted together with the saccharopine standard, whereas the pipecolate standard was eluted ≈ 0.1 min later. Specific assays for cystine and pipecolate by LC/MS/MS indicated that these amino acids did not account for the saccharopine peak, and that their concentration (cystine: 0.51 ± 0.03 and 0.63 ± 0.29 nmol/g and pipecolate: 2.0 ± 1.0 and 1.5 ± 0.7 nmol/g, respectively for *l2hgdh* ^*+/+*^ and *l2hgdh* ^-/-^ mice; mean values ± SEM for n = 3) was not increased in the *l2hgdh* ^-/-^ mice compared to the *l2hgdh* ^*+/+*^. There were no statistically significant changes in the concentration of amino acids in the plasma and in testis (Table 1).

**Table 1:** Amino acids in brain, testis and plasma of *l2hgdh* ^*+/+*^ and *l2hgdh* ^*−/−*^ mice.

	Brain		Testis		Plasma	
	*l2hgdh* ^*+/+*^	*l2hgdh* ^*−/−*^	*l2hgdh* ^*+/+*^	*l2hgdh* ^*−/−*^	*l2hgdh* ^*+/+*^	*l2hgdh* ^*−/−*^
Samples (n)	10	6	4	4	3	3
Alanine	667 ± 118	535 ± 71	1244 ± 116	1102 ± 171	293 ± 76	268 ± 18
Saccharopine	16 ± 3	< 1.0 *				
Aspartate	2868 ± 356	2105 ± 212	2027 ± 167	2275 ± 419	9 ± 3	10 ± 1
Glutamate	8693 ± 593	6825 ± 334*	3102 ± 552	3084 ± 262	33 ± 12	46 ± 12
Phenylalanine	53 ± 8	59 ± 7	68 ± 12	77 ± 11	92 ± 17	94 ± 28
Glycine	1216 ± 128	954 ± 192	2540 ± 79	2719 ± 212	181 ± 24	210 ± 10
Histidine	50 ± 8	60 ± 9	63 ± 11	71 ± 10	55 ± 7	67 ± 17
Isoleucine	58 ± 13	53 ± 6	74 ± 9	84 ± 5	149 ± 34	146 ± 50
Leucine	107 ± 21	114 ± 10	160 ± 43	163 ± 5	310 ± 123	315 ± 181
Lysine	187 ± 25	638 ± 93*	228 ± 33	260 ± 31	329 ± 152	395 ± 177
Methionine	25 ± 6	26 ± 7	34 ± 7	40 ± 7	49 ± 9	45 ± 3
Asparagine	94 ± 11	81 ± 10	143 ± 15	165 ± 33	61 ± 9	55 ± 14
Glutamine	5923 ± 713	3205 ± 433	1282 ± 66	1424 ± 281	695 ± 79	443 ± 125
Arginine	87 ± 5	200 ± 27*	66 ± 12	60 ± 6	97 ± 20	114 ± 18
Serine	811 ± 52	550 ± 124*	278 ± 12	275 ± 74	107 ± 17	107 ± 25
Threonine	309 ± 30	379 ± 43	280 ± 50	315 ± 67	153 ± 17	180 ± 63
Valine	215 ± 62	138 ± 11	470 ± 67	469 ± 36	363 ± 202	367 ± 226
Tryptophan	nd	nd	18 ± 1	26 ± 4	36 ± 16	47 ± 31
Tyrosine	41 ± 12	45 ± 12	61 ± 16	63 ± 8	75 ± 29	86 ± 44
P-Serine	86 ± 15	82 ± 17	154 ± 23	148 ± 17	11 ± 4	13 ± 4
Taurine	8874 ± 524	8763 ± 553	2508 ± 185	2528 ± 138	618 ± 194	705 ± 47
P-Ethanolamine	1714 ± 78	1659 ± 95	4810 ± 196	4910 ± 99	11 ± 4	16 ± 3
α-Aminoadipate	230 ± 50	136 ± 93	171 ± 41	175 ± 15	14 ± 2	16 ± 10
Cystathionine	26 ± 8	32 ± 7	nd	nd	nd	nd
β-alanine	76 ± 10	77 ± 10	nd	nd	nd	nd
GABA	3069 ± 243	2769 ± 97	33 ± 3	31 ± 4	nd	nd
Ammonia	845 ± 240	937 ± 145	278 ± 13	276 ± 44	190 ± 57	257 ± 39
Carnosine	135 ± 39	109 ± 9	nd	nd	nd	nd
Ethanolamine	100 ± 20	174 ± 14*	158 ± 33	176 ± 50	nd	nd
α-Aminobutyrate	29 ± 7	18 ± 5	13 ± 2	15 ± 6	29 ± 10	18 ± 2
Ornithine	11 ± 2	10 ± 1	17 ± 5	13 ± 2	67 ± 28	112 ± 62
Citrulline	9 ± 1	14 ± 4	16 ± 6	12 ± 1	49 ± 18	54 ± 30

Same experimental procedure as in Fig. 5.

Values are means ± SEM for the number of samples indicated that are shown in nmol/g (brain, testis) or nmol/ml (plasma).

*Significantly different values (alpha < 0.0016) are indicated with an asterisk.

The analysis was performed with the Student t-test with a Bonferroni correction for multiple testing (32 parameters).

### Presence of lysine-α-ketoglutarate reductase in brain and its inhibition by L-2-hydroxyglutarate

Lysine degradation is usually assumed to take place via the formation of saccharopine in liver but via L-pipecolate in brain [28] (Fig. 4). The finding that in brain lysine accumulation was accompanied by a suppression of saccharopine (Fig. 5A; Table 1) suggested that lysine degradation could proceed in brain via saccharopine and that this pathway was inhibited by L-2-hydroxyglutarate.

**Fig 6:**
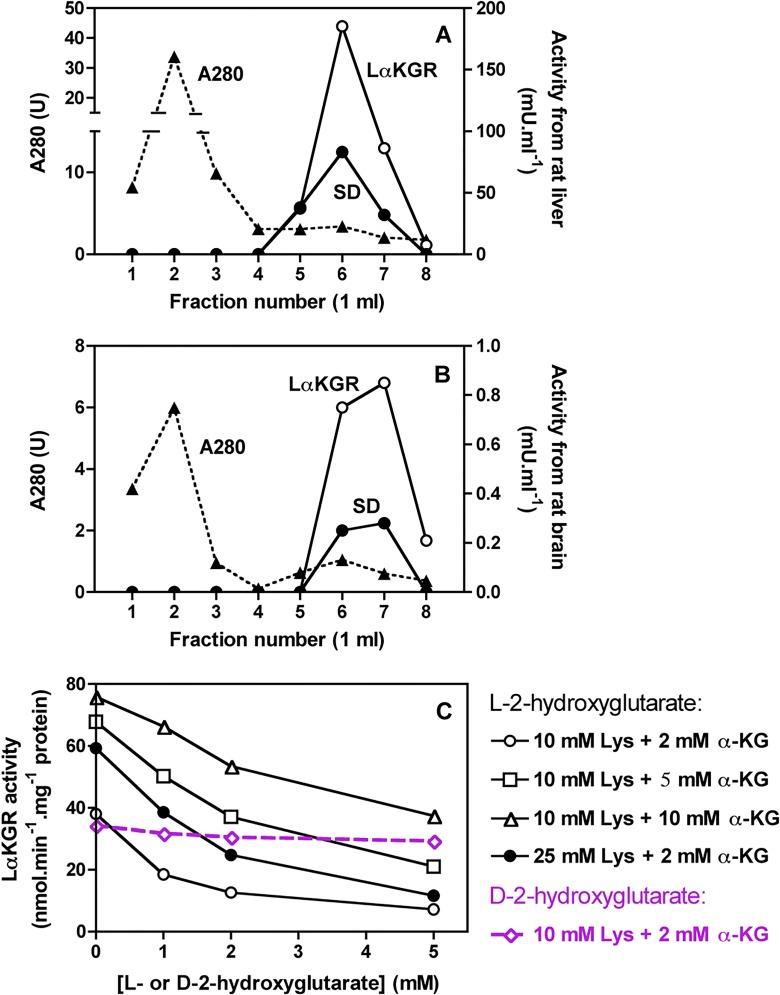
Presence of the bifunctional enzyme lysine-α-ketoglutarate reductase/saccharopine dehydrogenase in brain (A, B) and inhibition of lysine-α-ketoglutarate reductase by L-2-hydroxyglutarate (C). (A) and (B) show the elution profile of lysine-α-ketoglutarate reductase (LαKGR) and saccharopine dehydrogenase (SD) from Blue Trisacryl columns on which a mouse liver (A) or a mouse brain (B) extract have been applied. (C) Partially purified lysine-α-ketoglutarate reductase from mouse liver was assayed at 30°C in the presence of 25 mM Hepes, pH 8.0, 0.15 mM NADPH, 1 mM dithiothreitol, 10 mM lysine (Lys) and 2, 5 or 10 mM α-ketoglutarate (α-KG) as indicated and increasing concentrations of L-2-hydroxyglutarate. The effect of L-2-hydroxyglutarate was also tested in the presence of 25 mM lysine and 2 mM α-ketoglutarate and that of D-2-hydroxyglutarate with 10 mM lysine and 2 mM α-ketoglutarate.

We first checked for the presence of lysine-α-ketoglutarate reductase in brain by chromatographing extracts of mouse brain and liver on Blue Trisacryl columns, on which lysine-α-ketoglutarate reductase is well retained and eluted only with high salt concentrations (Fig. 6). We found indeed that brain, whether from control or *l2hgdh* ^-/-^ mice, contained an activity that was about 150-fold lower than the activity present in mouse liver. This activity co-eluted with saccharopine dehydrogenase both in the case of the liver and brain enzyme (Fig. 6A and 6B). This is expected since the two enzymes are known to form a bifunctional protein [28].

We next tested whether lysine-α-ketoglutarate reductase (purified from mouse or rat liver) would be inhibited by L-2-hydroxyglutarate and found indeed that this enzyme was inhibited by this compound (Fig. 6C), whereas almost no inhibition was observed with D-2-hydroxyglutarate. The inhibition by L-2-hydroxyglutarate was competitive with respect to α-ketoglutarate, with a Ki of 0.7 mM. Lysine-α-ketoglutarate reductase forms a bifunctional enzyme with NAD-dependent saccharopine dehydrogenase. This activity, which, expectedly, coeluted with lysine-α-ketoglutarate reductase, was not inhibited by L-2-hydroxyglutarate (not shown).

### Pathological analysis

**Fig 7:**
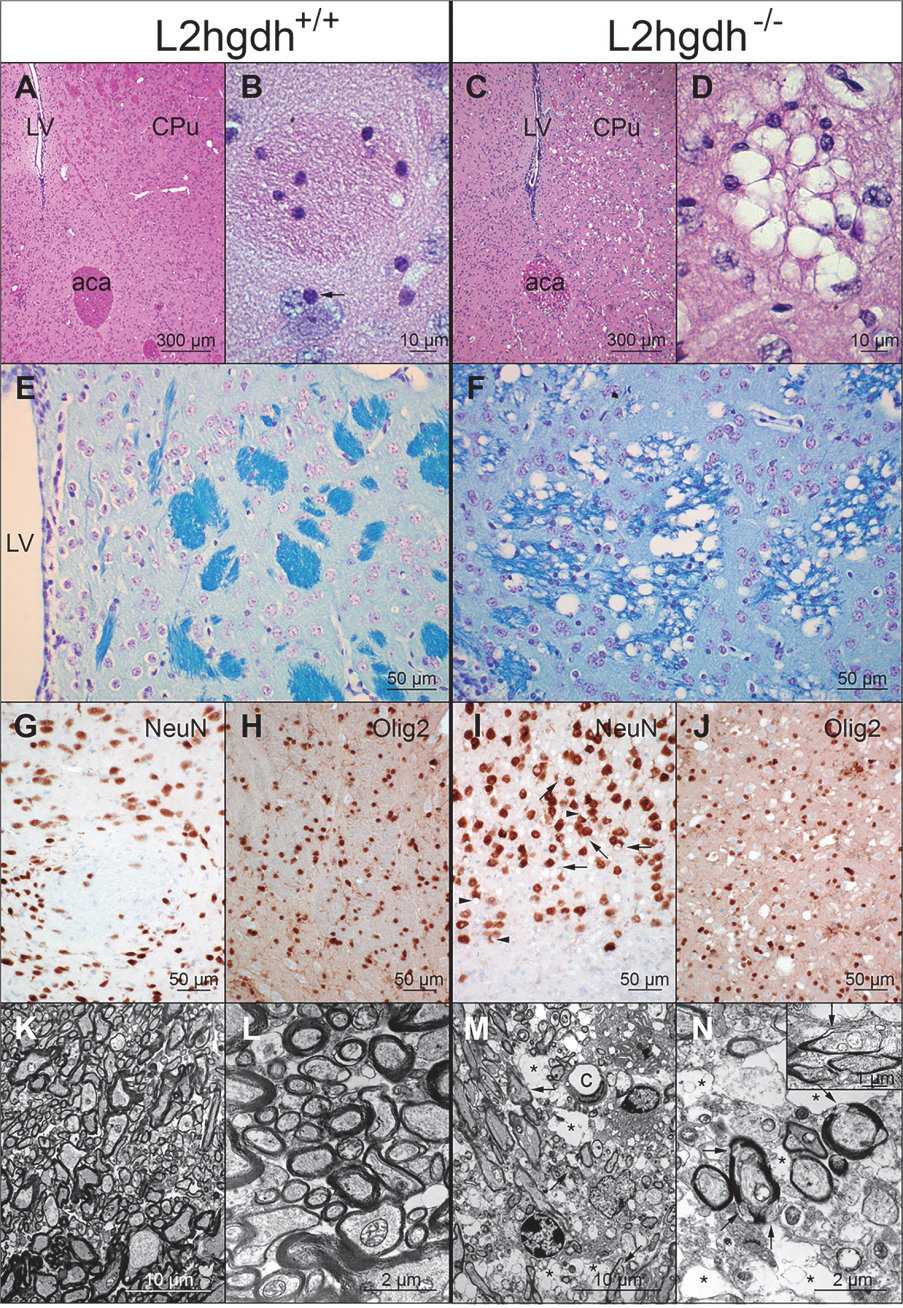
Pathological analysis of *l2hgdh* ^-/-^ mice. The figure compares the histological and ultrastructural appearance of the brain of *l2hgdh* ^*+/+*^ (left panels: A, B, E, G, H, K, L) and *l2hgdh* ^-/-^ mice (right panels: C, D, F, I, J, M, N). Hematoxylin and eosin staining shows low-magnification of the lateral part of the brain (A, C) at the level of the lateral ventricle (LV), the caudate putamen (striatum; CPu) and the anterior commissure, anterior part (aca). Higher magnification of pencils of Wilson are shown in panels B and D. Notice the satellite oligodendrocyte at B (arrow). Luxol fast blue staining of myelin is shown in E and F, highlighting the predominant presence of vacuoles in the pencils of Wilson of the striatum of the *l2hgdh* ^-/-^ mouse. Panels G-J are microphotographs from the junction between cortex and white matter in the vicinity of corpus callosum. Immunostaining of NeuN (G and I) shows that most vacuoles in the *l2hgdh* ^-/-^ mice are at distance from the nucleus of neurons (notice the abundance of vacuoles in the lower part of panel I, devoid of neurons) although a few vacuoles indent the nucleus of some neurons (arrowheads). However, the nucleus of satellite oligodendrocytes is also present in close contact to the vacuole indenting the nucleus of some neurons (arrows), preventing to identify clearly which cell type actually contains the peri-neuronal vacuole. In contrast, immunostaining of Olig2 (H and J) clearly shows that most vacuoles are in close contact to the nucleus of oligodendrocytes in the *l2hgdh* ^-/-^ mouse brain. Notice the small size of the nucleus of oligodendrocytes compared to the nucleus of neurons. Panels K-N are Transmission Electron Microscopy photographs of striatum tissue. Ultrastructural analysis (K-N) shows many empty-looking cell processes (asterisks in panels M and N), containing dilated cytoplasmic organelles, in the glia of *l2hgdh* ^-/-^ mice. The myelin sheath of many axons appears focally altered or disrupted (arrows at M and N), sometimes at the vicinity of empty-looking spaces. C, capillary lumen. The length of the scale bar is indicated in each panel.

**Fig 8:**
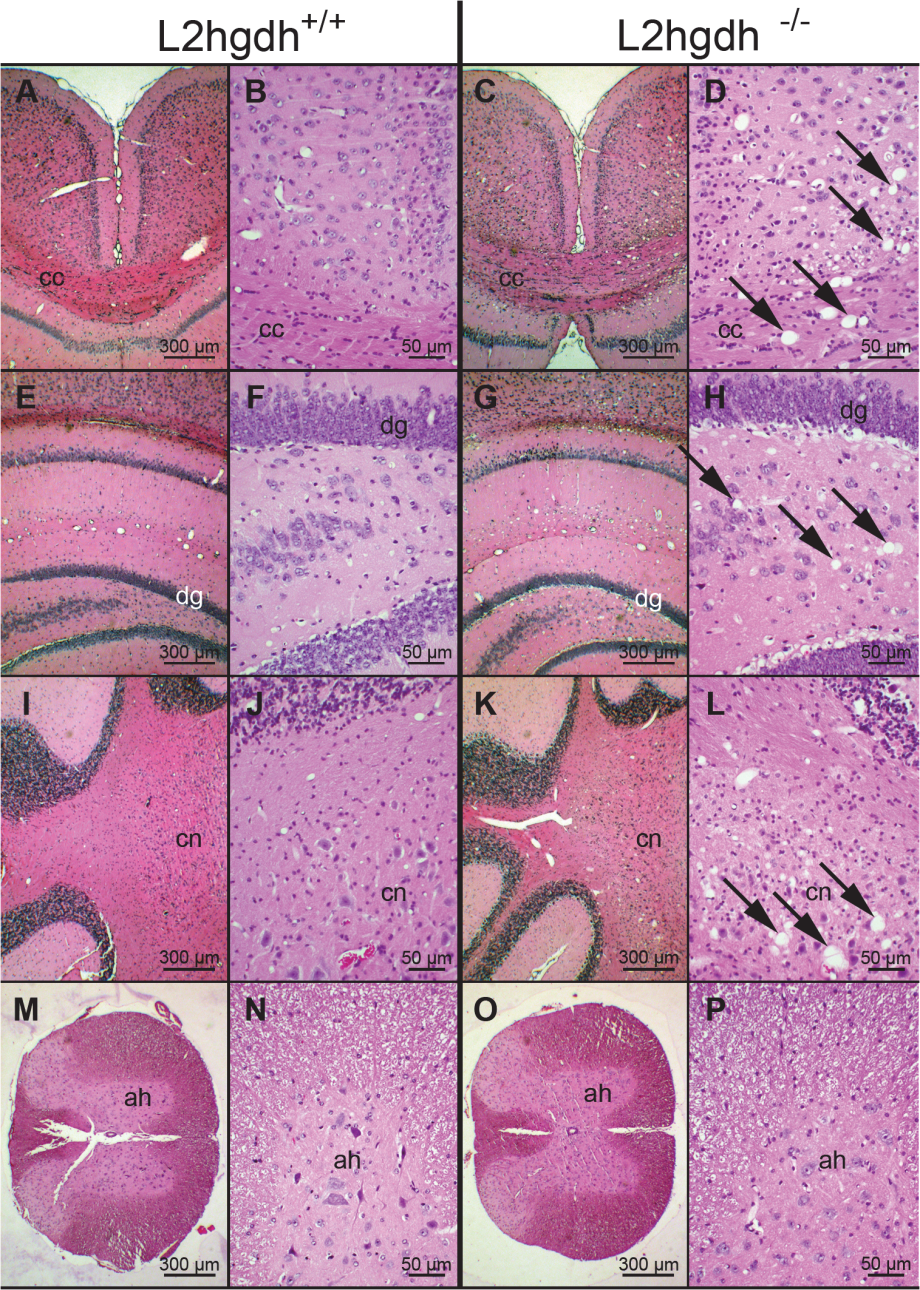
Representative microphotographs of different sites of the brain and spinal cord of *l2hgdh* ^-/-^ compared to *l2hgdh* ^*+/+*^ mice. A-D, transverse sections through the corpus callosum (cc); E-H, transverse sections through the hippocampus (dg, dentate gyrus); I-L, sections through the cerebellum and cerebellar nuclei (cn); M-P, transverse sections through the spinal cord (ah, anterior horn). Left panels (A, B, E, F, I, J, M, N) are representative of *l2hgdh* ^*+/+*^ mice and right panels (C, D, G, H, K, L, O, P) of *l2hgdh* ^-/-^ mice. On each side, the right panel is a higher magnification of the left panel, where arrows indicate lesions seen in brains of *l2hgdh* ^-/-^ mice. Scale bars correspond to 300 μm or 50 μm as indicated.

Pathological analysis on the brain of *l2hgdh* ^-/-^ mice was performed on 5–7 month-old mice. The brain of the *l2hgdh* ^-/-^ mice consistently showed an extensive spongiotic appearance that was predominant in the caudate putamen (Fig. 7C), the basal ganglia and the brainstem, in particular in the pencils of Wilson and the white matter fascicles, where the myelin bundles appeared distorted (Fig. 7D and 7F). The deep layers of the cerebral cortex and the corpus callosum (Fig. 8A-D) also showed a spongiotic pattern as well as the cerebellar nuclei (Fig. 8I-L). The hippocampus was less severely affected, with vacuoles in its white matter, mostly under the corpus callosum (Fig. 8E-H). In contrast, the spinal cord did not show a spongiotic pattern (Fig. 8M-P). At high magnification, the cytoplasm of oligodendrocytes, and possibly of astrocytes, appeared vacuolated (Fig. 7D and 7F). Most vacuoles were present in oligodendrocytes as assessed by immunostaining of NeuN for neurons and Olig2 for oligodendrocytes (Fig. 7I and J). The nucleus of some neurons was also indented by vacuoles but it was not possible to localize these vacuoles confidently inside the neurons since they could also be inside the cytoplasm of the satellite oligodendrocytes (Fig. 7I). Oil red O and Sudan black did not stain the vacuoles in frozen sections, indicating they did not contain lipids, and no mucin accumulation was disclosed by alcian blue staining of paraffin sections (not shown).

At the ultrastructural level, many empty-looking dilated cell processes that contained altered, often dilated, organelles (“vacuoles inside vacuoles” pattern) were observed in the glia of *l2hgdh* ^-/-^ mice (Fig. 7M and N). It was not possible to determine to which cell type these cytoplasmic processes belonged. The myelin sheath of axons often appeared disrupted, sometimes with spaces dissociating the myelin layers (Fig. 7N). Immunostaining of Ki67 did not indicate any cell proliferation and cleaved caspase-3 was not immunodetected, suggesting that apoptosis did not occur in the brain of *l2hgdh* ^-/-^ mice (not shown). There was no evidence for increased occurrence of tumors in brain or other organs in *l2hgdh* ^-/-^ mice.

### Locomotor and cognitive function

Locomotor activity in a familiar environment and consumption of food and drink were not significantly different between *l2hgdh* ^-/-^ and *l2hgdh* ^*+/+*^ mice. While muscle strength of fore- and hind-limbs were not significantly different, *l2hgdh* ^-/-^ mice appeared to be more susceptible to fatigue, as indicated by the wire test (Table 2). No impairment of coordination was observed during the accelerating rotarod test (data not shown).

**Table 2:** Psychomotor tests.

	*l2hgdh* ^*+/+*^	*l2hgdh* ^*−/−*^	p value
**Food intake (g/g body weight) (n = 6)**	0.256 ± 0.014	0.222 ± 0.020	0.185
**Drink intake (ml/g body weight) (n = 6)**	0.330 ± 0.045	0.237 ± 0.012	0.073
**Rearing (count/48h) (n = 6)**	1855 ± 810	4940 ± 927	0.031
**Horizontal activity (AU) (n = 6)**	7409 ± 1624	9344 ± 2752	0.558
**Wire test (s) (n = 14)**	85 ± 20	32 ± 9	0.024
**Fore and hind limb grip (g/g body weight) (n = 14)**	7.6 ± 0.4	7.2 ± 0.3	0.33
**Open field (n = 6)**			
Total distance moved (cm/20 min). 1^st^ test	4117 ± 565	7207 ± 1161	0.032
Total distance moved (cm/20 min). 2^nd^ test ^¥^	2111 ± 263	5947 ± 1210	0.011
Decrease (%)	46.2 ± 6.4	20.1 ± 6.9	0.020
**Y maze test (n = 6)**			
Alternance (%)	76.6 ± 4.6	55.0 ± 0.9	0.0009
Number of entry (count/5 min.)	17.2 ± 2.0	27.3 ± 2.8	0.014

Values are means ± SEM.

For all the tests, statistical analyses were performed using the Student t-test except for the open field test where statistical analysis was performed using a Two-way repeated measures ANOVA.

^¥^ The second test was done one month after the first one. Note that the decrease of alternance observed in *l2hgdh* ^*−/−*^ mice could not be explained by a decrease of activity, which was actually increased (number of entry).

Interestingly, in comparison to *l2hgdh* ^*+/+*^ mice, rearing activity was increased by about 2.6 fold in *l2hgdh* ^-/-^ mice (Table 2). The distance moved by the *l2hgdh* ^-/-^ mice when placed in a novel environment (open field test) was increased by about 175% compared to *l2hgdh* ^*+/+*^ mice (Table 2). Furthermore, in contrast to *l2hgdh* ^-/-^ mice, *l2hgdh* ^*+/+*^ mice progressively diminished their activity during the testing period (20 min), reflecting some habituation to their environment. Moreover, when the test was repeated after one month, *l2hgdh* ^*+/+*^ mice presented a locomotor activity that was diminished by half, whereas the activity of *l2hgdh* ^-/-^ mice was diminished by only 20%, again showing a decreased habituation to a known environment for *l2hgdh* ^-/-^ mice (Table 2).

**Fig 9:**
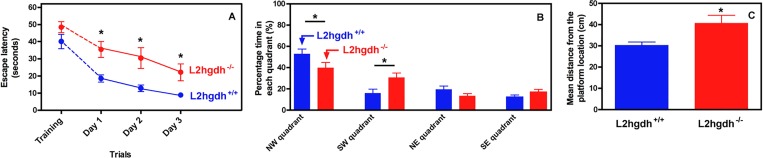
Morris Water maze test. The average escape latencies, (i.e., the time required for *l2hgdh* ^*+/+*^ and *l2hgdh* ^-/-^ mice to reach the platform) on the training day and on the 3 consecutive trial days are shown in (A). The percentage of the time the mice spent in each quadrant of the platform arena on the 5th day of testing (once the platform is removed) is shown in (B). The average distance (in cm) between the position of the mice during the latter test and the position where the platform had been previously placed (Gallagher's coefficient) is shown in (C). Values are expressed as the means ± SEM (n = 9). Statistical analyses were performed using a two-way repeated measures ANOVA (A), a one-way ANOVA (B) followed by a Newman-Keuls test, or a Student t-test (C). *: p < 0.05.

Learning ability of *l2hgdh* ^*+/+*^ and *l2hgdh* ^-/-^ mice was evaluated with the Y maze test and the Morris water maze test. In the Y maze test (Table 2), *l2hgdh* ^-/-^ mice displayed a significantly lower percentage of alternations compared with *l2hgdh* ^*+/+*^ mice, indicating an impairment of working memory. In the Morris water maze test (Fig. 9), the escape latency did not differ significantly between the *l2hgdh* ^*+/+*^ and *l2hgdh* ^-/-^ mice on the training day (when the platform was visible). The swimming speed was also similar, suggesting the absence of any major locomotor defect. It decreased progressively for both groups during the test trials performed during the following three days and became then significantly lower in the *l2hgdh* ^*+/+*^ mice compared to the *l2hgdh* ^-/-^ mice (Fig. 9A), indicating that the *l2hgdh* ^-/-^ mice had a reduced learning capacity. This conclusion was supported by the finding that during the probe test performed on the fifth day (the platform was then removed), the *l2hgdh* ^*+/+*^ mice spent more time than the *l2hgdh* ^-/-^ mice in the quadrant where the platform had been present (Fig. 9B), and swam, on average, closer to the platform than did the *l2hgdh* ^-/-^ mice during this test (Fig. 9C).

**Fig 10:**
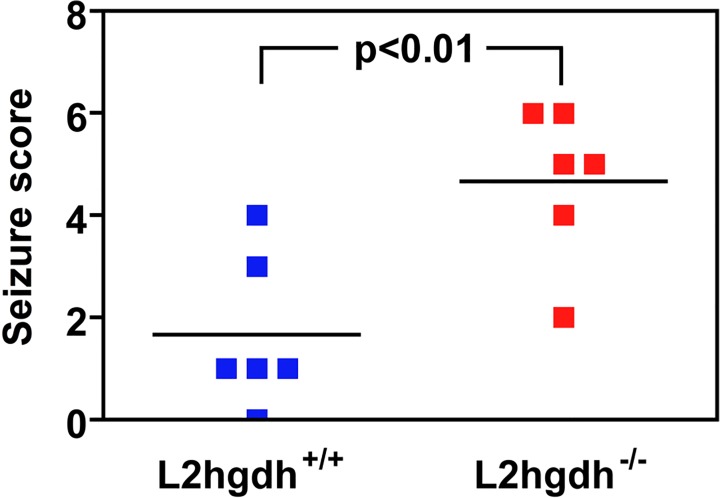
Sensitivity of *l2hgdh* ^-/-^ mice to the convulsant pentylenetetrazole. Six *l2hgdh* ^*+/+*^ and 6 *l2hgdh* ^-/-^ mice were placed in a plastic chamber (15 × 25 × 40 cm) and habituated for 10 min before intraperitoneal injection of a subconvulsive dose (40 mg/kg) of pentylenetetrazole (PTZ, Sigma-Aldrich, St. Louis, MO, USA). They were then video-tracked for 20 min and their behavior was classified and scored as follows: 0: normal; 1: immobilization; 2: facial, vibrissal and forelimb clonus (short myoclonic jerk); 3: myoclonic jerking consisting of a whole body jerk with or without irregular, bilateral forelimb movements; 4: generalized clonic seizures with kangaroo posture; 5: generalized tonic–clonic seizures with loss of posture tone; 6: death (Watanabe et al. 2013). Statistical analyses were performed using the Student t-test (p = 0.0062).

Finally, the sensitivity of *l2hgdh* ^-/-^ mice to convulsants was assessed by intraperitoneal injection of a subconvulsive dose of pentylenetetrazole. In contrast to *l2hgdh* ^*+/+*^ mice, this dose was sufficient to induce major convulsive seizures in *l2hgdh* ^-/-^ mice (Fig. 10).

## DISCUSSION

### Source of L-2-hydroxyglutarate

Previous work based on enzyme purification indicated that the main enzyme able to produce L-2-hydroxyglutarate in liver is mitochondrial L-malate dehydrogenase [8], a widespread enzyme present in all tissues. This conclusion was consistent with data obtained with fibroblasts of patients with L-2-hydroxyglutaric aciduria, indicating that L-2-hydroxyglutarate is formed from the mitochondrial pool of α-ketoglutarate rather than from the cytosolic pool [29]. We now confirm these conclusions by showing that the formation of L-2-hydroxyglutarate in MEF cells is dependent on the activity of mitochondrial L-malate dehydrogenase. The majority of the L-2-hydroxyglutarate formed results therefore from a metabolic error, i.e. the formation of a non-classical metabolite catalyzed by the side reaction of an enzyme that has a different physiological function.

The finding that male mice excrete significantly more L-2-hydroxyglutarate than female mice and that L-2-hydroxyglutarate is particularly elevated in testis indicate that in this tissue, L-2-hydroxyglutarate may be largely formed by LDHC (also known as LDHX). LDHC, which is exclusively expressed in sperm cells, is known to be rather non specific, since it is able to reduce not only pyruvate, but also α-ketoglutarate and other α-keto acids to the corresponding L-2-hydroxyacids [9, 30]. The role, if any, of L-2-hydroxyglutarate and other L-2-hydroxy acids that are also presumably formed by LDHC is not known. Yet the importance of LDHC is underlined by the fact that its absence leads to male sterility or hypofertility in mice [27]. No evidence for hypofertility was, however, observed when male and female *l2hgdh* ^-/-^ mice were crossed (not shown), suggesting that L-2-hydroxyglutarate metabolism does not play a critical role in testis physiology. Since LDHC is also highly expressed in human testis (as indicated by the BioGPS database), it is likely that L-2-hydroxyglutarate accumulates to high levels also in human testis. Whether this has any clinical consequence is unknown.

### Mechanism of lysine accumulation

Lysine metabolism classically proceeds in mammals either via the saccharopine pathway or the L-pipecolate pathway, which converge to form aminoadipate semialdehyde (Fig. 4) [28]. The first pathway, which is present in liver, kidneys and, with lower activities, in most other tissues, involves reduction of lysine and α-ketoglutarate to saccharopine followed by oxidation of the latter to L-glutamate and α-aminoadipate semialdehyde. These two activities are catalysed by one single bifunctional enzyme, whose deficiency results in hyperlysinemia [31]. α-Aminoadipate semialdehyde is then oxidized by aminoadipate semialdehyde dehydrogenase (ALDH7A1) to aminoadipate, which is then further metabolized via α-ketoadipate.

The second pathway, which is thought to be the main one in brain, is initiated by the conversion of lysine to its α-keto derivative by an unidentified enzyme, possibly an oxidase or a transaminase. Piperideine-2-carboxylate, which results from the spontaneous cyclisation of the product of the first reaction is then reduced to L-pipecolate, by imine reductase, an enzyme that is essentially present in brain, and which was recently identified as the product of the CRYM (μ-crystallin) gene [32]. Pipecolate is then oxidized to piperideine-6-carboxylate (Fig. 4).

The concentration of lysine is higher in the plasma and in the CSF of patients with L-2-hydroxyglutaric aciduria [33]. We reproduced this observation for brain in mice with L-2-hydroxyglutaric aciduria. The increase is particularly impressive, since it reached about 4-fold. A non significant trend to an increase is also found in blood.

The finding that lysine-α-ketoglutarate reductase is inhibited by L-2-hydroxyglutarate, a structural analog of one of the two substrates of this enzyme, with a Ki that is lower than the concentration of L-2-hydroxyglutarate in brain suggests that there is a metabolic block at the level of this enzyme. This is also supported by the finding that the concentration of saccharopine is decreased in the brain of mice with L-2-hydroxyglutaric aciduria. Lysine-α-ketoglutarate reductase is known to be active in mouse brain at early stages of development [34]. Its presence in the adult brain is disputed, since it was not found by Sauer et al. [35], but well in the present study, in agreement with previous mRNA expression data [36]. Taken together with the metabolite modifications found in L-2-hydroxyglutaric aciduria, the low activity of the bifunctional enzyme (about 0.7% of the activity found in liver) suggests that lysine metabolism, which is slow in brain, proceeds at least partly via saccharopine.

Intriguingly, no significant difference in plasma or testis lysine concentration were observed between control and *l2hgdh* ^-/-^ mice. In plasma, this is likely due to the fact that the L-2-hydroxyglutarate concentration is much less elevated in liver, the main organ responsible for lysine degradation, than it is in brain. In testis, this is possibly due to the absence of lysine-α-ketoglutarate reductase in this tissue, but this point has not been explored.

Kamoun et al. [33] proposed that the hyperlysinemia found in L-2-hydroxyglutaric aciduria is due to a depletion in the concentration of α-ketoglutarate, as they noted that the concentration of lysine is decreased in metabolic diseases where the concentration of α-ketoglutarate is increased. As we see here, an additional mechanism is the direct effect of L-2-hydroxyglutarate on lysine-α-ketoglutarate reductase.

The effect of L-2-hydroxyglutarate accumulation on lysine metabolism is interesting from the biochemical standpoint, but does probably not contribute to the neurological symptoms, since defects in lysine metabolism due to lysine-α-ketoglutarate reductase deficiency do not appear to cause pathological symptoms [37].

We have no simple explanation for the change in the concentration of other amino acids in brain. The lack of difference in amino acid concentrations in testis of wild-type and knockout mice, despite high levels of L-2-hydroxyglutarate in this tissue in the L-2-hydroxyglutarate dehydrogenase-deficient mice, is possibly due to higher exchange rates of the amino acid pools between testis and the circulation, compared to brain.

### Origin of the brain lesions

In humans, L-2-hydroxyglutaric aciduria usually presents as a slowly progressing neurodegenerative process manifested by mental retardation, spasticity and ataxia, and seizures appearing during infancy, and classically induces pathognomonic MRI changes including signal abnormalities of the subcortical cerebral white matter, putamen, caudate nucleus, globus pallidus and dentate nucleus [38, 39, 40]. However, phenotypic variations have been reported such as a rapidly progressing disease in a neonate [2] or on the other hand a poorly symptomatic disease diagnosed in a 40 year-old male [41]. The latter case did not show dentate nucleus MRI abnormalities. Only a few pathological analyses have been reported. The seminal report by Larnaout et al. [38] confirmed the extensive demyelination and cavitation of the white matter, mainly in the subcortical regions, spongiosis of the globus pallidus and the dentate nucleus with marked cell loss, dilation of ventricles, moderate basal ganglia atrophy, and subcortical necrotic foci in all cerebral lobes of a 30 year-old male with advanced disease. Another pathological analysis of the brain of a 15 year-old patient with advanced disease confirmed symmetric cystic cavities in the peripheral subcortical areas with intense spongiosis of the neuropil and of the subcortical white matter, but no ventricular dilation, a better preserved cerebellar white matter, and relatively few abnormalities of the basal nuclei and the dentate nuclei, although spongiosis was also present in the brainstem and around the dentate nuclei [40]. In contrast, the brain of the 1 month-old baby showed no spongiosis but severe atrophy, loss of neurons and gliosis of the brainstem and the cerebellum, lesions strikingly different from those reported in the other cases [2]. In dog, L-2-hydroxyglutaric aciduria also shows marked spongiform changes, but surprisingly, these affect predominantly the gray matter of the cerebral cortex, thalamus, brainstem and cerebellum [42]. In our mouse model, the extensive spongiosis of the white matter is similar to human lesions (except the neonate patient) but no cavitation is found. Spongiosis predominates in the pencils of Wilson and white matter fascicles surrounding the caudate putamen and basal ganglia but is also present in the subcortical white matter, in the deep part of the cerebral cortex, in the brainstem and in the dentate nuclei. The rest of the cerebellum and the hippocampus are minimally affected and no lesions are disclosed in the spinal cord.

The absence of stainable material in the vacuolated cytoplasm, in particular by oil red O and Sudan black on frozen sections, and the ultrastructural appearance of empty-looking dilated cellular processes suggest a hydropic degeneration of the cytoplasm of oligodendrocytes extending into the myelin sheath of axons, and possibly also of astrocytes and of some neurons. It should be noted that myelination was disrupted focally but well organized elsewhere, indicating that it was most likely correctly performed during brain development before local disruptions appeared during disease progression.

Spongiform encephalopathy is seen in different pathologies. In transmissible spongiform encephalopathies due to prions, vacuoles are found in neurons and in their processes, sparing the white matter and in particular the myelin bundles of the striatum [43]. In contrast, *l2hgdh* ^-/-^ mice show marked vacuolar changes of the white matter and lamellar splitting of the myelin sheath of axons. These lesions attributed to alterations in the oligodendrocytes are also seen in a model of leukoencephalopathy resulting from disruption of the chloride channel ClC-2 [44] and in the *Nur7* aspartoacylase-deficient mouse model of Canavan disease [43, 45]. However, distribution of the spongiform lesions varies among these three pathologies as well as among species.

The marked vacuolar changes of the white matter found in the *l2hgdh* ^-/-^ mice are reminiscent of Canavan disease but marginally involve the hippocampus and the cerebellum and not the spinal cord, in striking contrast to the spongy degeneration reported in the *Nur7* aspartoacylase-deficient mouse model of Canavan disease [45]. The spongy degeneration reported in the *Nur7* mouse model is extensive in the cerebellum, the hippocampus and the gray matter of the spinal cord in addition to the cerebral cortex, the subcortical white matter, the midbrain and the pons but not in the optic nerves nor in the corpus callosum [45].

In the leukoencephalopathy model caused by disruption of the chloride channel ClC-2, spongy degeneration predominates in the cerebellum, the corpus callosum, the internal capsule, the brainstem and the white matter of the spinal cord but is not found in the optic nerves [44]. ClC-2 appears to be expressed in oligodendrocytes and astrocytes, in addition to some neurons of the hippocampus. ClC2-deficient (*Clcn2* ^-/-^) mice display an early, severe retinal degeneration that results in almost complete blindness from birth on and the selective absence of vacuolation in their optic nerves suggests that the alterations of oligodendrocytes depend upon neuronal activity. Interestingly, *Clcn2* ^-/-^ animals also lacked overt neurological deficits and reduced seizure thresholds despite widespread and extensive vacuolation of the white matter [44]. This contrasts with the wide-based ataxic gait of young *Nur7* mice and the tremors and seizures that appear in these mice with age. These observations indicate that the extent of spongiform degeneration is not directly linked to the neurological symptomatology.

Leukodystrophy is a common feature of metabolic diseases in which a dicarboxylic acid accumulates in brain (Canavan disease, glutaric aciduria type I, L-2-hydroxyglutaric aciduria). This has been ascribed to poor permeability of the blood brain barrier to dicarboxylic acids [46]. We propose that the mechanism of the toxicity may additionally involve irreversible pumping of dicarboxylate into astrocytes and glial cells. Dicarboxylates, including L-2-hydroxyglutarate, are substrates for NaDC3 (SLC13A3), a sodium dependent transporter, which is expressed in glial cells, though not in neurons [47]. The citrate transporter (NaDC2) shows no affinity for L-2-hydroxyglutarate or other dicarboxylates [48, 49, 50]. It is therefore likely that L-2-hydroxyglutarate accumulates mostly in glial cells (astrocytes and oligodendrocytes) and therefore leads to major perturbations of these cells.

### Correlation of neurological symptoms with pathological lesions in mice

L-2-hydroxyglutaric aciduria is consistently accompanied by major neurological problems, such as progressive mental deficiency, severe cerebellar dysfunction, mild extrapyramidal and pyramidal symptoms, progressive macrocephaly, and seizures [51, 52]. The *l2hgdh* ^-/-^ mice also display neurological symptoms but the functional deficiencies appear limited compared to the extensive spongiform degeneration of the white matter, suggesting that neurological symptoms could be linked to other lesions, poorly or not visible morphologically. The increased locomotor activity observed in the open field and in the Y maze tests might reflect the presence of lesions in basal ganglia. Associated to the lack of habituation observed in the open-field test, it could also be due to hippocampal defects, as it is known that hippocampal lesions produce hyperactivity associated with a lack of habituation to a novel environment [53]. Moreover, hippocampal lesions are also associated with short-term and long-term learning deficits. Accordingly, we observed a deficit of short-term memory (Y maze) and a deficit of long-term memory (Morris water maze, long-term habituation to open field environment). Finally, *l2hgdh* ^-/-^ mice exhibited a more pronounced susceptibility to the convulsive drug pentylenetetrazole, an effect also observed in animals presenting hippocampal lesions [54].

### Conclusion

The concept of metabolite repair implies that abnormal metabolites need to be repaired because the abnormal products that may accumulate under some conditions may be damaging. In this respect it is important to identify (1) the ‘culprit’ enzyme; (2) the metabolite repair enzyme; (3) the targets on which the abnormal metabolite acts. This paper provides new information about all three questions and opens perspective for the elucidation of pathophysiological mechanisms.
